# Chromatographic Purification of Lithium, Vanadium, and Uranium from Seawater Using Organic Composite Adsorbents Composed of Benzo-18-Crown-6 and Benzo-15-Crown-5 Embedded in Highly Porous Silica Beads

**DOI:** 10.1021/acsomega.2c02427

**Published:** 2022-07-27

**Authors:** Yu Tachibana, Tomasz Kalak, Masahiro Tanaka

**Affiliations:** † Department of Nuclear System Safety Engineering, Graduate School of Engineering, Nagaoka University of Technology, 1603-1, Kamitomioka-machi, Nagaoka-shi, Niigata 940-2188, Japan; ‡ Department of Industrial Products and Packaging Quality, Institute of Quality Science, Poznań University of Economics and Business, Niepodległości 10, Poznań 61-875, Republic of Poland; § National Institute for Fusion Science, 322-6, Oroshi-cho, Toki-shi, Gifu 509-5292, Japan

## Abstract

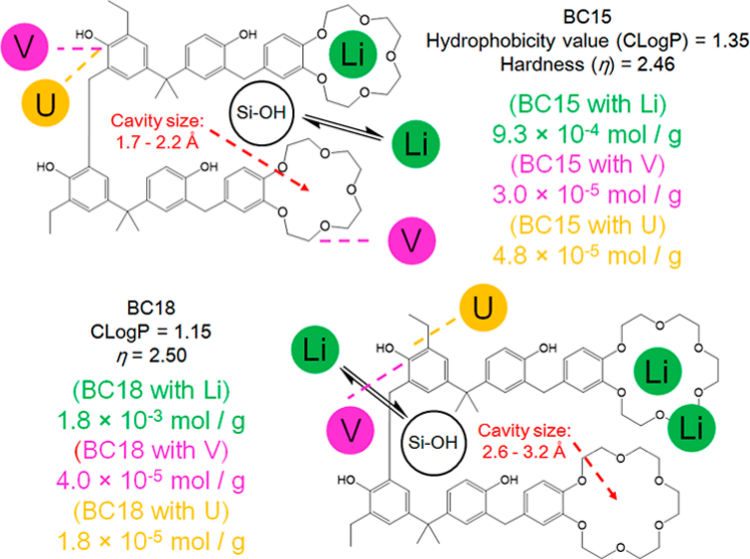

The use of the composite adsorbents composed of benzo-15-crown-5 (abbreviated as BC15) and benzo-18-crown-6 (BC18) for the simultaneous recovery of vanadium (V), uranium (U), and lithium (Li) from seawater has been proposed for industrial applications. The adsorption and desorption behavior of these elements on BC15 and BC18 has been examined in various types of aqueous solutions over a wide temperature range. As a result, it was shown that BC15 and BC18 have sufficient adsorption ability for the simultaneous recovery of V, U, and Li from seawater. Moreover, it was seen that the distribution coefficients (*K*_d_) of V decrease with an increase in [HCl]_T_ (subscript T: total concentration), indicating that the anionic V species such as H_2_V_4_O_13_^4–^ are exponentially changed into the cationic V species such as V^3+^, VO^2+^, and VO_2_^+^ under the condition [HCl]_T_ = 1.0 M, and the complexation reactions between BC15 (or BC18) and the initial V structures are inhibited. Besides, it was reasonably shown that the adsorption mechanism is the path through the electrostatic interaction between the anionic V species such as H_2_V_4_O_13_^4–^, and the −C–O–C– single bond that the electron density is eccentrically located in ether functional groups in crown ether rings in BC15 and BC18 (or the −C–OH single bond that the electron density is eccentrically located in bisphenol A in BC15 and BC18). Then, the chromatography experiment of V, U, and Li on BC15 (or BC18) at 298 K was carried out by flowing seawater, 1.0 × 10^–2^ M HCl, and 1.0 M HCl in sequence. The first peak of V can be separated from the plateau of Li and the first and second peaks of U in the case of the BC15 system. The recovery ratios of V and U were more than 80%. On the other hand, entirely overlapping chromatograms were obtained in the case of the BC18 system, and accordingly, the recovery ratios of V and U were much lower. In short, the separation efficiency of V with BC15 is more pre-eminent than that with BC18. Judging from these results, the durability of BC15 was finally assessed for industrial applications, that is, the aforementioned chromatography experiment was repeatedly carried out to check whether V, U, and Li were stably and mutually separated from seawater or not. The evidence that the recovery performances of V, U, and Li from seawater do not decrease at all after at least five cycle tests was provided. This indicates that this information will be valuable for the development of a practical chromatographic technology to simultaneously recover V, U, and Li from seawater.

## Introduction

Lithium (Li) is today recognized as one of the most essential minerals in the modern world. However, it is very difficult to effectively extract Li from the environment due to technical and economic issues. In addition, the industrial demand for Li is now growing year by year. Basically, the variety of industrial applications leads to an increase in the demand for Li, for instance, Li_2_CO_3_ in secondary batteries, glass ceramics, cement, and aluminum, LiOH in grease, CO_2_ absorption, mining, and pH adjusters in pressurized water reactors, Li metal in primary batteries, pharmaceuticals, and aluminum alloys, elastomers, another pharmaceutical, and *n*-butyllithium in agrochemicals, and Li specialties in electric materials and other pharmaceuticals and agrochemicals.^1−4^ Especially, from the viewpoints of environmental preservation and the reduction of CO_2_ emissions in recent years, it has been imperative to introduce zero-emission vehicles, including electric automobiles and portable electric devices equipped with reusable batteries. According to the latest literature, the proportion of the total consumption of Li used for Li secondary batteries was ca. 44% in 2017 and it will probably be ca. 65% in 2025.^5^ Even on taking the growing trend in the use of electric vehicles in the period from 2015 to 2050 into account, the total consumption of Li will reach approximately 5.11 million tons.^6^ Recently, the identified land-based total Li resources have substantially increased from about 14 million tons in 2019 to about 89 million tons in 2022, thanks to continuous exploration.^6,7^ In short, for the total land-based Li resources this year, the current total consumption occupies only about 6%. However, a huge amount of Li-6 may be required for nuclear fusion generation in the near future because Li-6 is used as a raw material for tritium (T) production in nuclear fusion reactors using the ^6^Li (n, α) T reaction.^3^ Due to T breeding from Li-6, the absolute amount of Li will certainly be reduced and its nuclear reaction makes it physically impossible to reuse Li-6 for other industrial applications. In order to achieve a better balance between supply and demand with the growing availability of fusion-based power plants, it will be required to create novel and cost-effective Li recycling technologies and/or to use other Li resources.

The natural Li resources used in our modern societies are mainly found in salt lakes. As typical salt lakes, there are Hombre Muerto Salt Lake, Olaroz Salt Lake, Rincon Salt Lake, and so forth in the Argentine Republic (resource: ca. 19 million tons), Atacama Salt Lake, and so forth in the Republic of Chile (resource: ca. 9.8 million tons), and Uyuni Salt Lake and so forth in the Republic of Bolivia (resource: ca. 21 million tons).^7^ The production among these accounted for at least 30% of the world’s Li production in 2021. Incidentally, oceans containing enormous amounts of Li (total oceanic abundance: approximately 231 billion tons) have a greater potential to become a promising natural Li resource, and the ratio of total oceanic abundance to mineral reserves on land is about 5.63 × 10^4^.^8^ However, the Li concentration in seawater is about 178 ppb [(25.6 μM (M = mol/L, L = dm^3^)] and its concentration is very low, compared with those of other major elements: sodium (10,800 ppm), magnesium (1,290 ppm), calcium (411 ppm), potassium (392 ppm), and so forth.^8^ Moreover, the oceanic Li resources are not formally counted as the amount of Li reserve as no commercial Li recovery technologies currently exist. To accumulate Li effectively in seawater, the Li recovery processes using cation-exchange reactions and/or complexation reactions, combined with the effect of steric hindrances have been thoroughly investigated.^3,5,9,10^ Owing to the introduction of the simple adsorption and desorption reactions using the above chemical behavior, the required plant engineering design can be simplified. Apropos, the solvent extraction process and yet another unique Li recovery process could be one of the promising methods.^5^ For example, the solvent extraction process using LIX54 (main component: α-acetyl-*m*-dodecylacetophenone) and trioctyl phosphine oxide (often called TOPO) or using Cyanex 923 consisting of four trialkylphosphine oxides and LIX54 has good extraction ratios for Li even in seawater.^11,12^ However, their kinetic performances are low or the extractants are expensive. In many cases, the selectivity, capacity, kinetics, and structural stability of adsorbents, including cation exchangers and Li extractants, are not suitable in seawater, and especially, most inorganic adsorbents dissolve gradually into seawater over a long immersion time.^3^ This means that adsorbents and extractants which maintain well-balanced relations with higher selectivity, capacity, and structural stability are required.

Uranium (U) is a common radioactive element in the environment and is slowly released when using U-containing phosphate fertilizers,^13^ natural deposits and discharge from postflotation waste,^4^ and combustion of coal and other fossil fuels.^14^ U accumulates in the kidneys and bones through drinking, eating, and inhalation, and its accumulation causes pathogenic effects such as cancer.^14^ According to the latest report, the identified total U resources are 6,147,800 tons and the global percentages for U resources by country in 2019 are 28% in the Commonwealth of Australia (1,692,700 tons), 19% in the Republic of Kazakhstan (906,800 tons), and 15% in Canada (564,900 tons).^15^ Moreover, more than 60% of the world’s mining production, which exists mainly as uraninite, comes from Kazakhstan, Australia, and Canada in 2020.^15^ With the current global U consumption rate, it is contemplated that the U reserves for nuclear electricity generation may be exhausted in about 80–120 years.^16^ U resources are well recognized as the low-carbon source of base load power generation. However, the current world energy requirements have the potential to be a twofold increase by the high annual growth of emerging economies by 2050 and the world population will exceed 13 billion by a high population growth rate by the same year.^16^ Hence, it is planned that the nuclear-generated electric power supplies some of the increased energy to eliminate this power shortage. In analogy with the Li resources, it is required to exploit new U resources. Seawater contains U, and its average concentration is about 1.39 nM.^8^ In short, the total abundance of seawater will become around 4.29 billion tons, which means that the ratio of total abundance of seawater to mineral reserves on land is about 7.84 × 10^2^ to 1.65 × 10^3^.^8^ Besides, the U resources correspond approximately to the U consumption of the global nuclear reactors over 13,000 years.^17^ According to the late-breaking data, the chemical forms of U(VI) in seawater with pH ≑ 8.2 are as follows: UO_2_(CO_3_)_3_^4–^, Mg[UO_2_(CO_3_)_3_]^2–^, Ca[UO_2_(CO_3_)_3_]^2–^, Ca_2_[UO_2_(CO_3_)_3_] (aq), (UO_2_)_11_(CO_3_)_6_(OH)_12_^2–^, UO_2_(OH)_2_ (aq), UO_2_(CO_3_)_2_^2–^, and (UO_2_)_2_CO_3_(OH)_3_^–^ and this means that U in seawater exists as neutral and anionic species.^3,16,18,19^

As with U recovery from seawater, many researchers have attempted to collect economically U in seawater using the following various types of adsorbents: (1) inorganic materials (inorganic adsorbents (IAs) [adsorption capacity (AC)/(mg of U/g of adsorbent) = 6 × 10^–3^ to 8.50 × 10^2^ (in water), = 7 × 10^–3^ (in seawater)],^20,21^ nanostructured carbons (NCs) [AC = 6 × 10^–3^ to 1.932 × 10^3^ (in water), = 2 × 10^–3^ to 3.4 (in seawater)],^22−24^ and mesoporous silica adsorbents [AC = 8 × 10^–3^ to 2.77 × 10^2^ (in water), = 3 × 10^–2^ to 1 × 10^–1^ (in seawater)^25−27^]; (2) organic materials (radiation-induced graft polymerization-prepared adsorbents (RIGPAs) [AC = 3 × 10^–3^ to 9.24 × 10^2^ (in water), = 6 × 10^–4^ to 5 (in seawater)]^28−33^ and atom-transfer radical polymerization-prepared adsorbents (ATRPAs) [AC = 2 –1.79 × 10^2^ (in water), = 6 × 10^–1^ to 5.2 (in seawater)],^34−37^ polymer adsorbents prepared by neither RIGPs nor ATRPs (PAs) [AC = 1.7 × 10^–2^ to 5.50 × 10^2^ (in water), = 4.2 × 10^–3^ to 2.81 × 10^1^ (in seawater)^38−41^]; (3) inorganic–organic hybrid materials (metal–organic frameworks (MOFs) [AC = 0 to 8.40 × 10^2^ (in water)],^42,43^ covalent organic frameworks (COFs) [AC = 5.0 × 10^1^ to 2.11 × 10^2^ (in water)],^44,45^ and porous-organic polymers (POPs) [AC = 4.0 × 10^1^ to 3.04 × 10^2^ (in water), = 2 (in seawater)^46,47^]; (4) biomaterials [proteins engineered genetically for high affinity and their connected materials (BMs)] [AC = 1 (mg-U/g-adsorbent) and 100 (pmol/bead) (in water), = 9.2 × 10^–3^ (in seawater)^48,49^]; and (5) waste materials [fly ash (FA): AC = 2.13 × 10^1^, slag (S): AC = 5.67 × 10^1^ (in seawater)].^4^ These summarized data imply that all adsorption reactions between U and their adsorbents are significantly blocked by other substances in seawater. Consequently, the development of adsorbents with higher selectivity for oceanic U recovery has still been worked energetically. However, it seems to be so difficult to overcome the economical challenge only in the viewpoint of selectivity. A change in thinking will be needed to provide a superior performance at a lower cost toward industrial applications.

**Figure 1:**
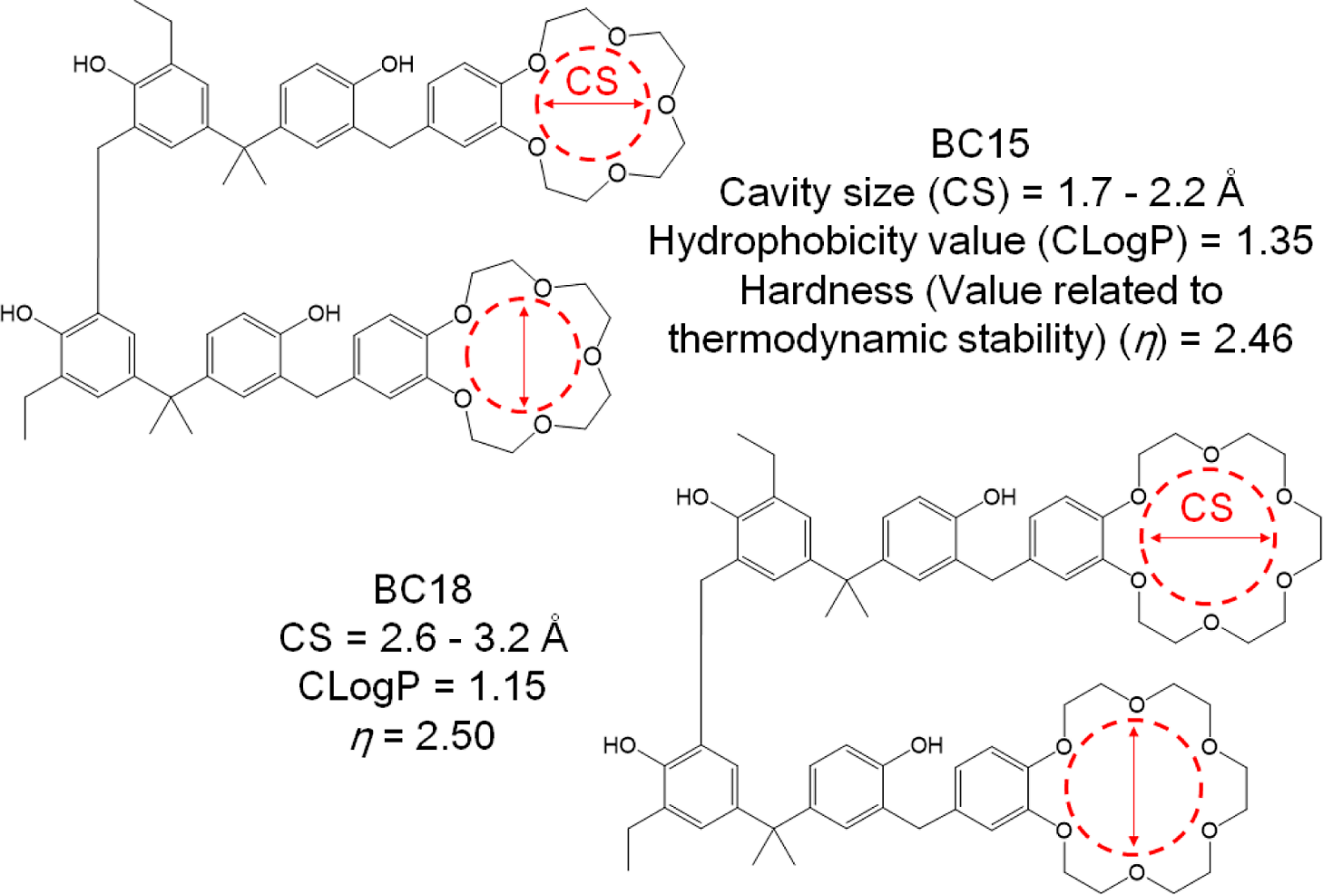
Cavity sizes, hydrophobicity values, and η values of synthesized BC15 and BC18. These parameters were calculated by using three kinds of software (ChemBioOffice Ultra 14.0, Cambridge Soft Corporation; General Atomic and Molecular Electronic Structure System (GAMESS); DV-*X*α MO calculation)^67−70^ and referred from the literature.^71^

As one of the means for solving this problem, we have proposed to simultaneously recover Li, U, and vanadium (V) from seawater by chromatography using previously synthesized adsorbents: benzo-15-crown-5 (abbreviated as BC15) and benzo-18-crown-6 (BC18), which have two kinds of functional groups (see Figure 1). The ether oxygen in the crown ether can selectively combine Li, compared with the results of other crown ethers.^3,50^ (UO_2_)_11_(CO_3_)_6_(OH)_12_^2–^ can be adsorbed against the hydroxyl group in bisphenol A in seawater.^3^ The higher adsorption ability of BC15 and BC18 for [Li(H_2_O)_*n*_]^+^ (*n* = 3–4) was confirmed, and their adsorption behavior is substantially attributable to their cavity sizes and degrees of hydrophobicity.^3,50^ On the other hand, the calculated distribution coefficients (*K*_d_) of BC15 and BC18 were (1.15 ± 0.17) × 10^3^ and (1.70 ± 0.08) × 10^3^, respectively, meaning that most of the U species contained in seawater under the experimental condition move to the surfaces of BC15 and BC18.^3,50^ As another element to recover simultaneously from seawater, V was chosen because it is often used as an additive for iron and steel production, a catalyst for chemical industry, a hydrogen storage material, a redox flow battery, a vanadium alloy for aircraft, dental implants, nuclear materials, and so forth, indicating that it is one of academically and commercially interesting valuable elements.^51−55^ The global V consumption has increased by approximately 45% compared with that of 2011, that is, it reached 102.1 kilotons in 2019, and moreover, the consumption is estimated to become 130.1 kilotons by the end of 2024.^56^ The global V resources exceed 63 million tons in 2022. Although the resources do not run dry immediately, the ratio of total abundance of seawater to mineral reserves on land is about 190, suggesting that the collection of V from seawater is reasonable.^8^ In the meantime, it has been well known that the U and V have a similar adsorption behavior against the surfaces of adsorbents and the interfaces of extractants, making it difficult to separate them mutually.^37,57−59^ However, the undue focus on the highest adsorption selectivity for U and V may cause their peak tailing in a chromatogram. On the other hand, the chemical forms of U and V change in a very dramatic way in a wide pH range. The key is to achieve the mutual separation of Li, U, and V from seawater using any durable materials here.

Based on these backgrounds, the adsorption and desorption mechanisms in the chromatographic purification of Li, U, and V have been examined and determined using BC15 and BC18, which are embedded in highly porous silica beads in aqueous solutions (seawater, 1.0 × 10^–2^ M HCl, and 1.0 M HCl), combined with the additional data by batch experiments over a wide temperature range. Then, with respect to industrial applications, the preceding chromatography experiment was repeatedly carried out to check the durability of the selected adsorbent.

## Materials and Methods

### Materials

The real seawater was sampled from the Sea of Japan around Gokahama in Nishikan-ku, Niigata, Japan, on January 15, 2021, and the sampling spot was located at 37.784° north latitude and at 138.817° east longitude. HCl (conc.: 35.0 wt %) was purchased from Nacalai Tesque, Inc. The standard V solution whose concentration was 998 mg/L in 5 wt % HNO_3_ was made by FUJIFILM Wako Pure Chemical Corporation. The raw material of this V standard solution was NH_4_VO_3_. On the other hand, Li and U in seawater were directly used without any additives. The used reagents for analyses were of special pure grade. BC15 and BC18 synthesized in our previous work were prepared for this study.^3^ Highly porous silica beads (Mizusawa Industrial Chemicals, Ltd.) were used to suppress the swelling and shrinking behavior of BC15 and BC18, and this support can enhance the mechanical strength of BC15 and BC18. These factors are of importance for industrial applications.

### Sample Preparation

The sampled seawater contained some fine particles. This removal was carried out using a filter composed of hydrophilic glass fibers with binder resin (AP25, Millipore) before all experiments. The filter’s pore size, diameter, and thickness were 2.0 μm, 47, and 1.2 mm, respectively. The initial Li concentration was 120 ± 5 ppb, while the concentration range of U was ≤2.30 ppb. These concentrations were somewhat lower than those from refs (8) and (60). This may be brought about by the weather on the preceding day of sampling. The V salt in the glass beaker was prepared by the evaporation of the V dissolved in the mixture of HNO_3_ and HCl. This evaporation was carried out using an infrared heating and drying lamp (IR100/110V375WRH, Iwasaki) and a hot plate kept constant at around 373 K in combination. Then, seawater was added into the beaker including the dried V salt. In addition, the V salt was dissolved by using an ultrasonic apparatus (UC-0515, Tocho). Before using, this stock solution was filtered. The initial V concentration range was found to be 19.4–20.3 ppb in the batch experiments, while it became 14.2–29.6 ppb in the chromatography experiments. HCl solutions ranging in concentration from 1.0 × 10^–2^ to 1.0 M were prepared by mixing ultrapure water produced from an ultrapure water production system (Milli-Q Integral 3, Merck Millipore). The quality level of ultrapure water was the concentration of total organic carbon below 3 ppb and with the values of specific electrical resistance being more than 18.2 MΩ cm.

### Adsorption Experiments

The adsorption processes of V were examined by batch-wise techniques to determine the distribution coefficients (*K*_d_) using BC15 and BC18 in HCl solutions, whose concentration range was from 1.0 × 10^–2^ to 1.0 M and the apparent thermodynamic parameters in seawater were Δ*G*, Δ*H*, and Δ*S* in a wide temperature range. 0.50 g of BC15 and BC18 was individually added into vials made of polypropylene containing 1.0 × 10^–2^ to 1.0 M HCl or seawater. Each solution volume was 10 mL. The initial counter cation of the functional groups in BC15 and BC18 was changed with the H form by flowing the aqueous solution of 1.0 M HCl. A combination of a polypropylene vial and addition of 1 wt % HNO_3_ was chosen to minimize the unnecessary adsorption by hydrophobic interactions and ion–exchange reactions between materials and metal ions.^61^ The sample solutions in vials were steadily stirred while maintaining a constant temperature (temperature. = 278–338 K) by using a shaking water bath. This shaking time was set to 24 h, indicating that the adsorption equilibriums were sufficiently reached in our experimental conditions. BC15 and BC18 were physically separated from sample solutions by using a centrifuge separator (H-36α, Kokusan) whose operating time was 10 min at 3500 rpm. The concentration data of Li, U, and V were provided by using atomic absorption spectrometry (AAS) (AA-6200, Shimadzu) and inductively coupled plasma mass spectrometry (ICP/MS) (7700×, Agilent). For AAS and ICP/MS measurements, an aqueous solution containing 1 wt % HNO_3_ was used as a diluent. The level of confidence of the obtained *K*_d_, Δ*G*, Δ*H*, and Δ*S* was in the range of ±1σ.

### Chromatography Experiments

**Figure 2:**
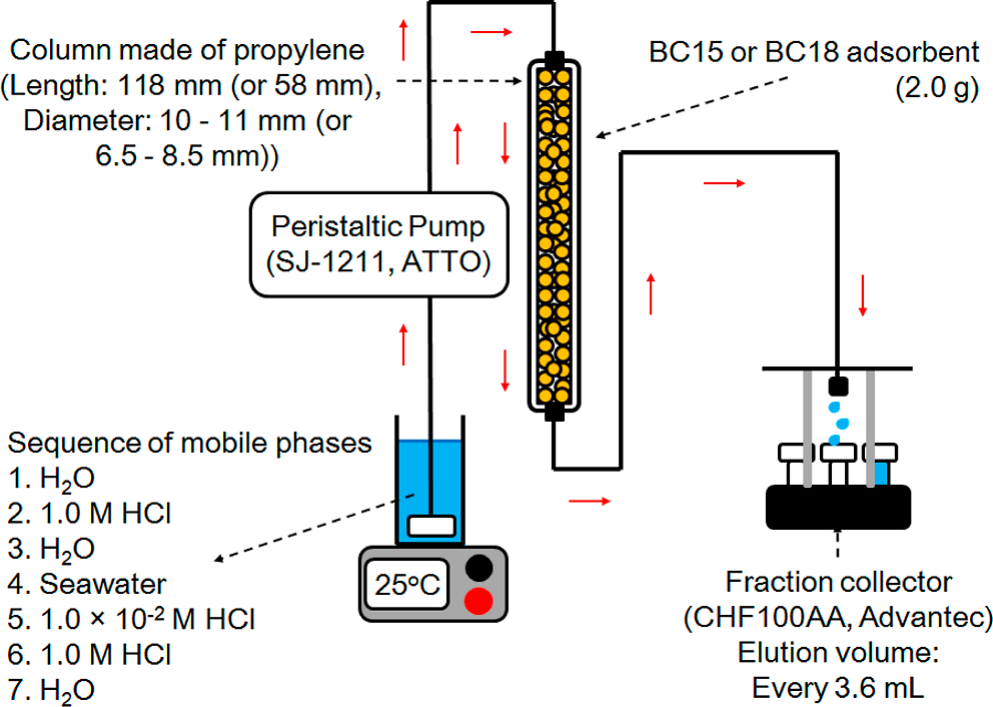
Chromatographic apparatus.

The chromatography experiments using plastic columns [Mini-column S-type (length (L): 58 mm, inner diameter (ID): 6.5–8.5 mm) and Mini-column L-type (L: 118 mm, ID: 10–11 mm), Muromachi Chemicals Inc.] were performed to evaluate the separation behavior of Li, U, V, and so forth and the V maximum adsorption capacities on BC15 and BC18. The internal temperature in these columns was controlled using air conditioners at 298 K. The columns were connected in series with a PharMed BPT tube of ID = 0.8 mm and outer diameter (OD) = 4 mm and a silicone rubber tube of ID = 3 mm and OD = 5 mm (see Figure 2). The embedded weight of BC15 and BC18 from which the weight of highly porous silica beads is excluded is 4.80 × 10^–1^ g for the V/BC15 system and 5.04 × 10^–1^ g for the V/BC18 system. The initial Na form of BC15 and BC18 was conditioned to the H form by using a 1.0 M HCl solution. After this conditioning, free H^+^ on BC15 and BC18 was removed by using H_2_O until the pH of column eluates became neutral. Next, column chromatography tests were performed using flowing seawater, 1.0 × 10^–2^ M HCl, and 1.0 M HCl in sequence. All flow rates were kept at 1.0 mL/min using a peristaltic tube pump (SJ-1211-II-H, Atto). Each volume of flowed mobile phases was 152 mL (seawater), 103 mL (1.0 × 10^–2^ M HCl), and 105 mL (1.0 M HCl) in the case of the V/BC15 system. In the V/BC18 system, each volume was 153 mL (seawater), 104 mL (1.0 × 10^–2^ M HCl), and 105 mL (1.0 M HCl). The 100 fractions including each 3.6 mL effluent were collected by using a fraction collector (CHF100AA, Advantech). The pH of each effluent was measured using a pH tester with 0.01 pH resolution and a precision of 0.01 pH (HI 98100, Hanna). The concentrations of Li, U, V, and so forth were measured using the AAS and the ICP–MS analyzers. The determination of their dead volumes was done by measuring the Na concentration in the collected effluents. As a diluent for these measurements, the same solution prepared in the adsorption experiments was used. The surface structures of BC15 and BC18 were observed using a scanning electron microscope (VE-8800, Keyence).

## Results and Discussion

### Adsorption Behavior of V on BC15 and BC18 in HCl Solutions

**Figure 3:**
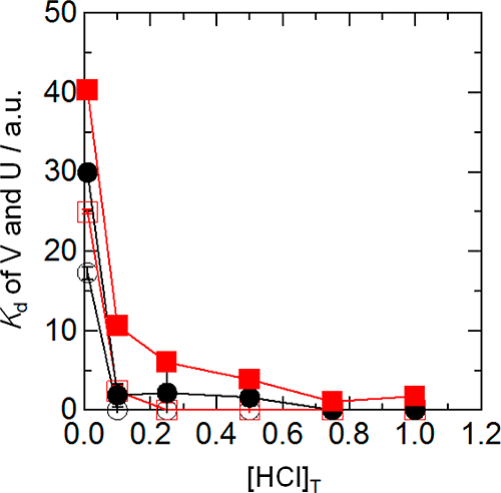
Plots of *K*_d_ vs [HCl]_T_. Temperature = 298 K. Particle size = 100–250 mesh, BC15 (or BC18) = 0.50 g, solution volume = 10 mL. ○: V with BC15, ●: U with BC15, □: V with BC18, ■: U with BC18 [the plots of *K*_d_ values of Li with BC15 (or BC18) were excluded because of the very low *K*_d_ values or no adsorption].

**Figure 4:**
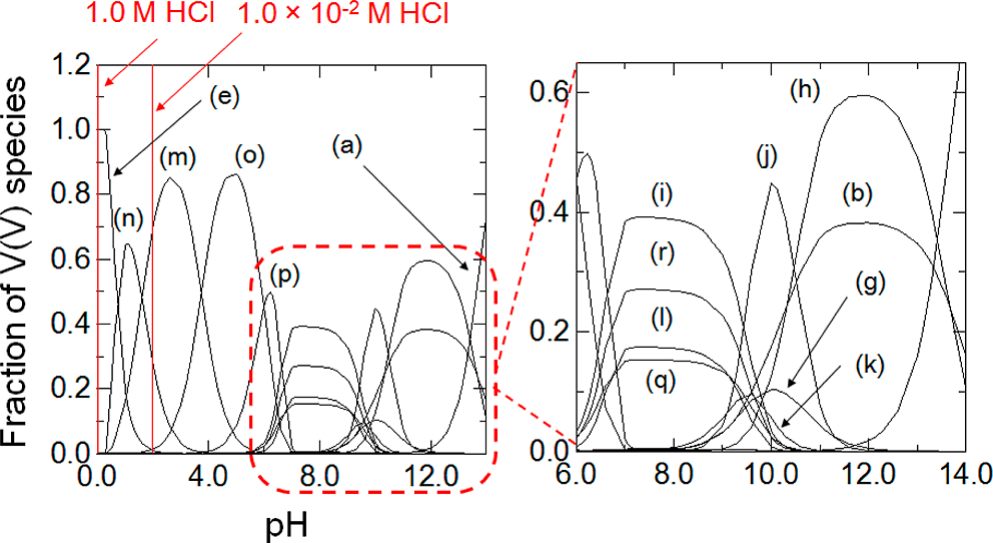
Distribution diagram of V(V) species as a function of pH at 298 K. The stability constants between VO_4_^3–^, VO_2_^+^, V_2_O_7_^4–^, V_4_O_13_^6–^, V_5_O_15_^5–^, V_10_O_28_^6–^, V_3_O_9_^3–^, V_4_O_12_^4–^, and H^+^ and H_2_O have been summarized by Smith and Martell in 1982 and 1989.^62,63^ (a) VO_4_^3–^, (b) HVO_4_^2–^, (c) H_2_VO_4_^–^, (d) H_3_VO_4_, (e) VO_2_^+^, (f) H_2_V_2_O_7_^2–^, (g) HV_2_O_7_^3–^, (h) V_2_O_7_^4–^, (i) H_2_V_4_O_13_^4–^, (j) V_4_O_13_^6–^, (k) HV_4_O_13_^5–^, (l) V_5_O_15_^5–^, (m) H_2_V_10_O_28_^4–^, (n) H_3_V_10_O_28_^3–^, (o) HV_10_O_28_^5–^, (p) V_10_O_28_^6–^, (q) V_3_O_9_^3–^, and (r) V_4_O_12_^4–^. With respect to the fraction of other species, (c) H_2_VO_4_^–^, (d) H_3_VO_4_, and (f) H_2_V_2_O_7_^2–^are excluded because of the small fraction of their V(V) species. Ionic strength = 0.5 M.

The adsorption equilibrium experiments were done using batch-wise techniques in order to evaluate the *K*_d_ values between V and BC15 (or BC18) in solutions ranging in concentration from 1.0 × 10^–2^ to 1.0 M at 298 K. These *K*_d_ values were calculated using the following eq 1.
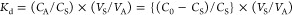
1where *C*_0_, *C*_S_, *C*_A_, *V*_S_, and *V*_A_ are the initial concentration of V in a HCl solution, the concentration of V in a HCl solution at adsorption equilibrium, the concentration of V on BC15 (or BC18) at adsorption equilibrium, the volume of the solution, and the volume of BC15 (or BC18), respectively. As a result, it was found that the *K*_d_ values of V decrease with an increase in [HCl]_T_ (subscript T: total concentration), as shown in Figure 3. This phenomenon indicates that the chemical species of V were dramatically changed and the complexation reactions between BC15 (or BC18) and V were canceled or the cation–exchange reactions between H^+^ and V preferentially proceeded by adding HCl. Therefore, the chemical species of V in a solution were examined in detail. V can exist with the oxidation states of +II, +III, +IV, and +V in a solution. V^2+^ formed by dissolving VO(c) can occur in nonoxidizing acids.^61^ V(III) from V_2_O_3_(c) can be present as V^3+^, VOH^2+^, and VO^+^ in an acid solution, while V_2_(OH)_2_^4+^ can be prepared from VCl_3_·6H_2_O (total concentration of V(III) and [V(III)] > 6 × 10^–3^ M in 1 M NaCl).^61^ It can be considered that the formations of green V^3+^ in an acidic solution and VO^+^ in a basic solution are dominant over the other V(III) species, judging from their stability constants (see Figure S1). The +IV oxidation state of V can be designed by using VO_2_ in acidic and basic solutions. V(IV) in aqueous solutions can mainly consist of monomeric, dimeric, and tetrameric structures and their hydrolysis products, relying on the V(IV) concentration and the pH value. In acidic solutions, the simplest formula of V(IV) is blue VO^2+^ but is not V^4+^. Then, the hydrolysis of VO^2+^ occurs to yield monomeric VOOH^+^ and dimer (VOOH)_2_^2+^. Besides, the formations of more complicated spices such as HV_2_O_5_^–^ (or VO_4_^4–^) and V_4_O_9_^2–^ were speculated in nearly neutral aqueous solutions.^61^ In the pH region using this condition, it was found that the main form is HV_2_O_5_^–^ until pH ≑ 2, and V_4_O_9_^2–^ is formed at a pH greater than 2 (see Figure S2). V(V) is contained in the two most familiar compounds, V_2_O_5_ and NH_4_VO_3_. The solubility of orange V_2_O_5_ into H_2_O is comparatively lower and the color of V_2_O_5_ in an aqueous solution becomes light yellow. Smith and Martell have summarized the stability constants between VO_4_^3–^, VO_2_^+^, V_2_O_7_^4–^, V_4_O_13_^6–^, V_5_O_15_^5–^, V_10_O_28_^6–^, V_3_O_9_^3–^, V_4_O_12_^4–^, and H^+^ and H_2_O at the ionic strengths (I) from 0 to 1.0 at 298 K.^62,63^ Seawater has an I value of 0.7.^64^ Hence, the forms of V(V) in seawater were predicted by using the stability constants related to the conditions *I* = 0.5 and temperature = 298 K, as shown in Figure 4. It can be pondered that V(V) exists as a complex mixture of monomeric and polymeric ions in properties, depending on the pH and total V(V) concentration. The typical redox reactions among these V species are as follows.^65^
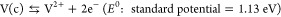
2
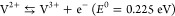
3
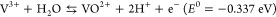
4
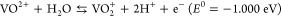
5

The formation of V^3+^ from V(c) and V^2+^ in eqs 2 and 3 may be reasonable in an aqueous solution. VO^2+^ and VO_2_^+^ will potentially be in a basic aqueous solution. In addition, the V salt obtained by evaporation of a 5 wt % HNO_3_ solution including NH_4_VO_3_ is used as a starting material. Hence, it can be regarded that the oxidation state of V species in seawater is pentavalent. This view is in accord with that by Isshiki.^60^ H_2_V_4_O_13_^4–^, V_4_O_12_^4–^, V_5_O_15_^5–^, and V_3_O_9_^3–^ mainly formed in seawater with pH = 7.3–7.7 (see Figure 4), while VO_2_^+^ existed in an acid aqueous solution ([HCl]_T_ = 1.0 M). V^3+^ and VO^2+^ can be produced via the foregoing redox reactions (eqs 4 and 5) under the condition [HCl]_T_ = 1.0 M (see Figures S1 and S2). When [HCl]_T_ was 1.0 × 10^–2^ M, the *K*_d_ value of V was high, meaning that H_2_V_10_O_28_^4–^ and H_3_V_10_O_28_^3–^ can coordinate with BC15 (or BC18). However, in the case of [HCl]_T_ = 1.0 M, the *K*_d_ value of V was very low, indicating that the crown ether rings of BC15 (or BC18) cannot catch V^3+^, VO^2+^, and VO_2_^+^ in a 1.0 M HCl solution. In a word, the predicted reasonable mechanism is the electrostatic interaction between H_2_V_10_O_28_^4–^ (or H_3_V_10_O_28_^3–^) and the single bond between the carbon atom with a higher positive charge and the oxygen atom with a higher negative charge triggered by the electronegativity in hydroxyl groups in bisphenol A in BC15 (or BC18) when [HCl]_T_ was 1.0 × 10^–2^ M. This estimation was similar to that of our previous result of U(VI).^3^ The desorption behavior of V on BC15 (or BC18) is not based on the cation–exchange reactions between H^+^ and V due to the addition of HCl as follows (see eq 6).
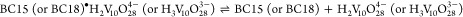
6

**Figure 5:**
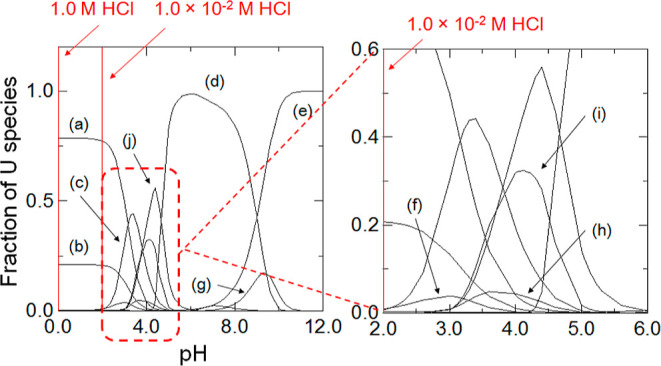
Distribution diagram of U(VI) species as a function of pH at 298 K. The stability constants between UO_2_^2+^ and H_2_O, Cl^–^, CO_3_^2–^, and CO_2_ (g) are reported by Grenthe in 2004.^19^ (a) UO_2_^2+^, (b) UO_2_Cl^+^, (c) (UO_2_)_2_(OH)_2_^2+^, (d) (UO_2_)_11_(CO_3_)_6_(OH)_12_^2–^, (e) (UO_2_)_2_CO_3_(OH)_3_^–^, (f) (UO_2_)_2_(OH)^3+^, (g) UO_2_(CO_3_)_3_^4–^, (h) (UO_2_)_3_(OH)_4_^2+^, (i) (UO_2_)_4_(OH)_6_^2+^, and (j) (UO_2_)_3_(OH)_5_^+^. With respect to the fraction of other species, UO_2_Cl_2_, UO_2_OH^+^, UO_2_(OH)_3_^–^, (UO_2_)_3_(OH)_7_^–^, (UO_2_)_4_(OH)_3_^5+^, UO_2_CO_3_, UO_2_(CO_3_)_2_^2–^, and (UO_2_)_3_(CO_3_)_6_^6–^ are excluded because of their low fraction of U(VI) species. Ionic strength = 0.5 M. [CO_3_^2–^]_T_ = 2.4 × 10^–3^ M (Fujinaga et al., 2005).^60^ [Cl^–^]_T_ = 6.1 × 10^–1^ M. [U]_T_ = 4.2 × 10^–7^ M. [CO_2_(g)]_T_ = 8.9 × 10^–3^ M (Fujii, 2017).^72^ This calculation result was compatible with those in refs (3) and (4).

Furthermore, it can be considered that the desorption behavior of U on BC15 (or BC18) is the following mechanistic path (see eqs 7 and 8), based on the U chemical forms in a 1.0 × 10^–2^ M HCl solution (see Figure 5).
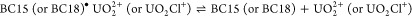
7
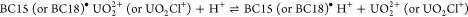
8

The difference between the *K*_d_ value of V and the *K*_d_ value of U in [HCl]_T_ = 1.0 × 10^–2^ M was larger than that in [HCl]_T_ = 1.0 M. This shows that the mutual separation of V and U(VI) using a chromatographic technique is possible.

### Chromatography of V on BC15 and BC18 in Aqueous Solutions

**Figure 6:**
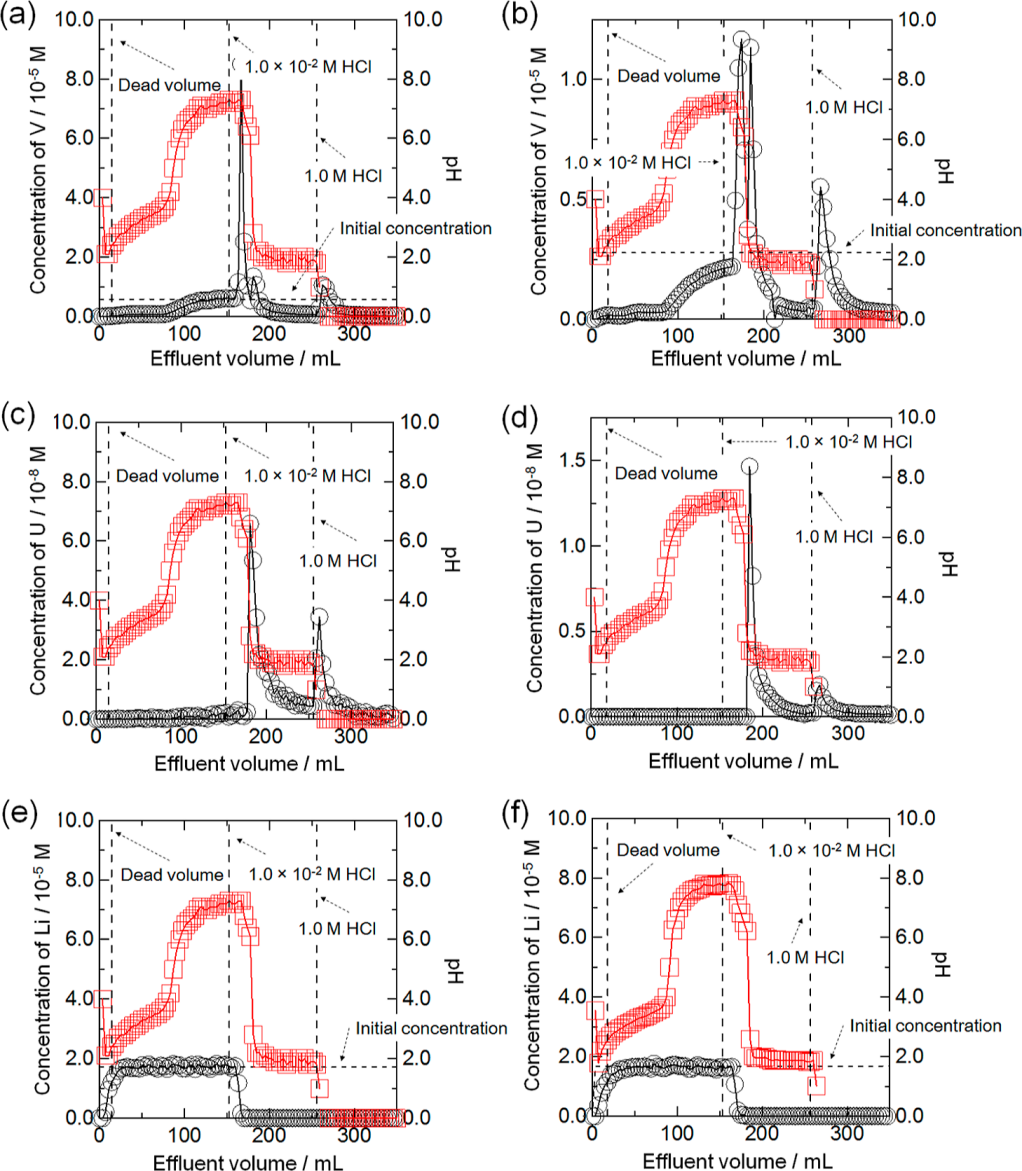
Chromatograms of V, U(VI), and Li(I) using BC15 and BC18 at 298 K. Particle size = 100–250 mesh. ○: Concentrations of V, U(VI), and Li(I) (black), □: pH value (red). (a) V with BC15. (b) V with BC18. (c) U with BC15. (d) U with BC18. (e) Li with BC15. (f) Li with BC18.

**Figure 7:**
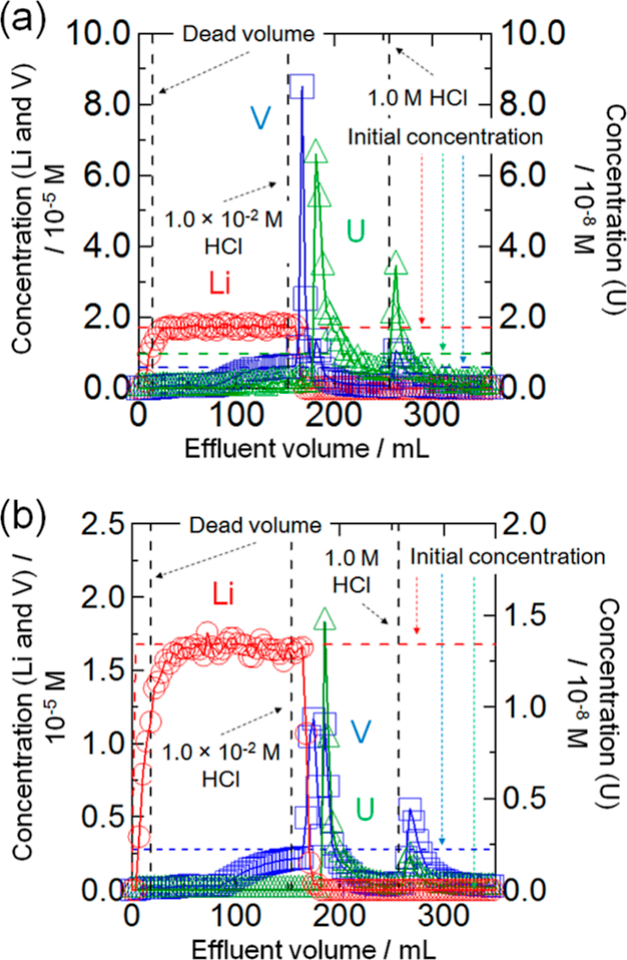
Chromatograms of Li(I), V, and U (VI) using BC15 and BC18 at 298 K. Particle size = 100–250 mesh. ○: Concentration of Li (red), □: concentration of V (blue), △: concentration of U (green). (a) Li, V, and U with BC15. (b) Li, V, and U with BC18.

The chromatographic concentration curves of V, U(VI), and Li(I) using BC15 and BC18 were given in Figures 6 and S3. It was found that the saturated concentrations of V are in accord with the initial ones. When BC15 and BC18 were used, the effluent volumes of saturated points for V/BC15 and V/BC18 systems were 134 and 103 mL, respectively. The dead volumes in the figures were 11 mL (Figure 6a,c,e), 12 mL (Figure 6b,d,f), and 5 mL (Figure S3). On the basis of the relationship between the initial concentrations of the feed solutions of V and these V curves, the maximum V adsorption capacities on BC15 and BC18 in seawater were calculated. The obtained maximum adsorption capacities are summarized in Table 1 to compare those reported previously. The V maximum adsorption capacities using BC15 and BC18 were 3.0 × 10^–5^ and 4.0 × 10^–5^ mol/g of adsorbent, respectively. BC15 and BC18 have much lower adsorption capacities for V than those of PAs by about 22 and 17 times, respectively. In addition, the adsorption capacity of PAs for U was also larger than those of BC15 and BC18 by about 3 and 7 times, respectively. Although PAs have promising adsorption capacities for the simultaneous recovery of V and U from seawater, it seems to be very difficult to selectively collect Li from seawater. The largest maximum adsorption capacity of U using slag (S) in the latest literature has been reported and its value is 56.7 mg/g of adsorbent.^4^ However, it can be easily expected that the amorphous structures of S and FA make it difficult to individually collect U from seawater. It can be considered that H_1.6_Mn_1.6_O_4_ with the largest maximum adsorption capacity of Li shows much lower adsorption ability for anionic V and U species in seawater. As shown in Figure 6a,b, the three kinds of peaks of V after flowing 1.0 × 10^–2^ M HCl and 1.0 M HCl in sequence were confirmed. In particular, the height of the second peak of V was the lowest among them in the case of BC15. The V recovery ratios of BC15 and BC18 were 101 and 93%, respectively. The maximum pH value was compatible with the initial pH value of seawater. The two kinds of U peaks were obtained after flowing the same two kinds of HCl solutions (see Figure 6c,d). The height of their second peaks was lower than those of the first peaks. The U recovery ratios were 109% (BC15) and 99% (BC18). The plateaus of Li concentrations were monitored and shown in Figure 6e,f. This means that the Li adsorption capacities of BC15 and BC18 were saturated. The Li recovery ratios of BC15 and BC18 were 101% and 99%, respectively. These concentration curves were merged (see Figure 7a), and it was found that the first peak of V separated from the plateau of Li and the first and second peaks of U. The V and U recovery ratios were 82 and 82%, respectively. On the other hand, the overlapped chromatograms were confirmed from the result of merged concentration curves (see Figure 7b), meaning that each recovery ratio of V and U was 37 and 0%, respectively. As a result, it was found that the separation efficiency of V with BC15 is more pre-eminent than that of BC18.

**Table 1:** Maximum Adsorption Capacities of V, U, and Li in Seawater Using Some Adsorbents

element	adsorbent	mg/g of adsorbent	mol/g of adsorbent	[refs]^a^
V	PAs	33.27	6.531 × 10^–4^	(41)
	BC15	1.5	3.0 × 10^–5^	this work
	BC18	2.0	4.0 × 10^–5^	this work
U	FA	21.3	8.95 × 10^–5^	(4)
	S	56.7	2.38 × 10^–4^	(4)
	PAs	28.1	1.18 × 10^–4^	(41)
	BC15	11	4.8 × 10^–5^	(3)
	BC18	4.2	1.8 × 10^–5^	(3)
Li	H_1.6_Mn_1.6_O_4_	40	5.8 × 10^–3^	(10)
	BC15	6.5	9.3 × 10^–4^	(3)
	BC18	12	1.8 × 10^–3^	(3)

a The references that the largest maximum adsorption capacities were reported were selected.

**Figure 8:**
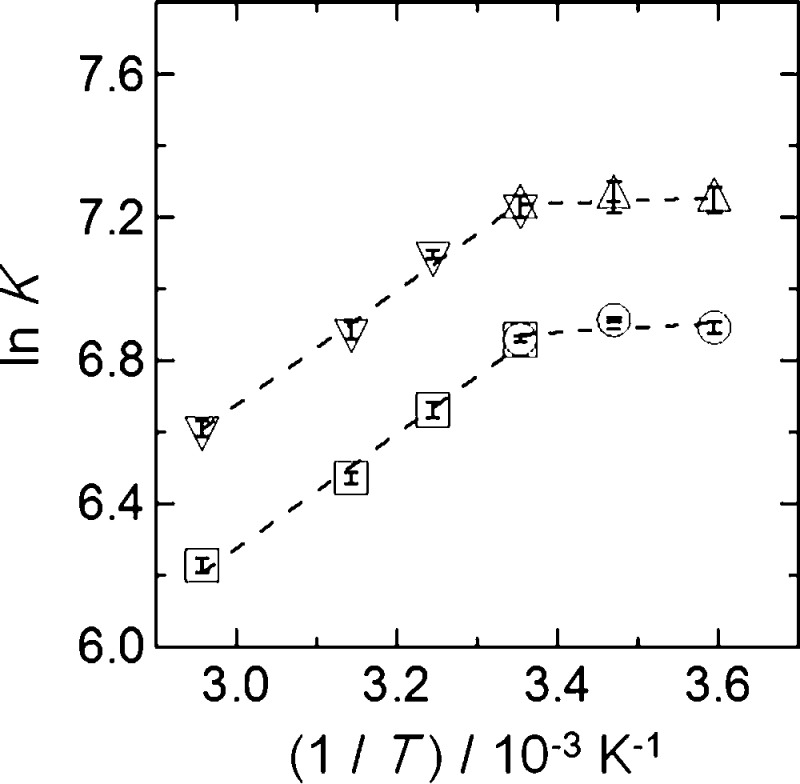
Plots of ln *K* values vs 1/*T* values. ○, □: BC15, △, ▽: BC18. Temperature = 278–338 K. Particle size = 100–250 mesh. BC15 and BC18 = 250 mg. Solution volume = 10 mL. pH = 4.3.

Therefore, the adsorption mechanisms between V and BC15 (or BC18) in seawater were examined in detail. Basically, the apparent Δ*H*, Δ*S*, and Δ*G* values for the adsorption of V on BC15 (or BC18) from the linear plots of ln *K* against (1/*T*) were calculated using the following eq 9 under the following condition: temperature = 278–338 K and final pH = 4.3 (see Figure 8). *K*_d_ was converted into *K*.^66^ Based on these data, the chemical surrounding of BC15 (or BC18) and V in seawater was analyzed.
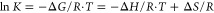
9where *R* and *T* represent the gas constant and absolute temperature. If the electrostatic interaction between V and the single bond between more positively charged carbon atoms bonded with hydroxyl groups in bisphenol A and its oxygen occurs, the plots of ln *K* versus (1/*T*) should be a straight line, indicating that one reaction proceeds. Nevertheless, the bent straight lines were observed in V/BC15 and V/BC18 systems, as seen in Figure 8. The observed phenomenon means that at least two kinds of adsorption mechanisms between V and BC15 (or BC18) occur under the conditions. The number of their adsorption mechanisms of V was in harmony with that of peaks of V confirmed in the chromatography experiments. The single bond between more positively charged carbon atoms in ether functional groups in crown ether rings and its oxygen and the single bond between more positively charged carbon atoms bonded with hydroxyl groups in bisphenol A and its oxygen interact with V in seawater.

The values of Δ*H*, Δ*S*, and Δ*G* are given in Table 2. The negative Δ*G* values indicate the spontaneous processes between V and BC15 (or BC18) proceed in seawater. The nearly zero Δ*H* value in the V/BC15 (or BC18) system from 278–298 K implies that neither the endothermic or exothermic process clearly proceeds. On the other hand, the negative Δ*H* value proves the exothermic process between V and BC15 (or BC18) in the temperature range from 298 to 338 K. The ether functional group in crown ether rings in BC15 (or BC18) and large polynuclear complexes such as H_2_V_4_O_13_^4–^, V_4_O_12_^4–^, V_5_O_15_^5–^, and V_3_O_9_^3–^, which may have a lower solubility can be regarded as a higher hydrophobic structure. On the contrary, the hydroxyl groups in bisphenol A in BC15 (or BC18) are surrounded by plenty of water in seawater. Before the electrostatic interaction between H_2_V_4_O_13_^4–^ (or V_4_O_12_^4–^, V_5_O_15_^5–^, and V_3_O_9_^3–^) and the single bond between more positively charged carbon atoms in ether functional groups in crown ether rings and its oxygen (−C–O–C– single bond) [or the single bond between more positively charged carbon atoms bonded with hydroxyl groups in bisphenol A and its oxygen (−C–OH single bond)] results in seawater, the hydration shells of V and those in BC15 and BC18 should be broken. This means that energies at varying degrees of the dehydration processes are required. Additionally, the corresponding energy to stabilize the conformation of electrostatic interaction between V (H_2_V_4_O_13_^4–^, V_4_O_12_^4–^, V_5_O_15_^5–^, and V_3_O_9_^3–^) and the −C–O–C– single bond (or the −C–OH single bond) was emitted in adsorption processes. The hydrophobicity of the −C–O–C– single bond is much higher than that of the −C–OH single bond. The different amounts of energy required to break their hydration shells arise because of a less amount of hydrated water of the −C–O–C– single bond in crown ether rings. Hence, the total energy balance consumed and emitted from the electrostatic interaction between the −C–O–C– single bond (or the −C–OH single bond) and V corresponds to the Δ*H* values, indicating that the Δ*H* values are nearly zero and negative in V/BC15 and V/BC18 systems. In other words, it can be considered that the electrostatic interaction between the −C–O–C– single bond in BC15 (or BC18) and V preferentially worked from 298 to 338 K, whereas another electrostatic interaction between the −C–OH single bond in BC15 (or BC18) and V mainly proceeded from 278 to 298 K. The parameters of the interaction between the −C–OH single bond in BC15 (or BC18) and V (temperature = 278–298 K) were very similar to those between the −C–OH single bond in BC15 (or BC18) and (UO_2_)_11_(CO_3_)_6_(OH)_12_^2–^ (temperature = 278–298 K) (see Table 2). The positive values of Δ*S* mean that the randomness arises due to the destruction of hydration shells of V and the single bonds having −C–O–C– and −C–OH structures far superior to the behavior of the electrostatic interactions of the V on the surface of BC15 (or BC18). Moreover, the difference in the Δ*S* values obtained from the two kinds of adsorption mechanisms mainly comes from the degree of destructing the hydration shells of the V and −C–O–C– structure (or −C–OH structure) in BC15 (or BC18). On the other hand, the apparent Δ*H*, Δ*S*, and Δ*G* values between BC15 (or BC18) and V (or U) are different from those between BC15 (or BC18) and Li, as shown in Table 2, indicating that the crown ether rings and the dissociated hydroxyl groups can selectively catch Li.^3^

**Table 2:** Apparent Thermodynamic Parameters between Metal Ions (V, Li, and U) and BC15 (or BC18) in Seawater in the Temperature Range of 278–338 K

system	temperature range (278–298 K)	temperature range (298–338 K)
V + BC15	Δ*H* = −1 ± 2 kJ/mol	Δ*H* = −13 ± 1 kJ/mol
	Δ*S* = 53 ± 6 J/(mol·K)	Δ*S* = 12 ± 2 J/(mol·K)
	Δ*G* = −17.0 ± 0.1 kJ/mol	Δ*G* = −17.0 ± 0 kJ/mol
V + BC18	Δ*H* = −1 ± 1 kJ/mol	Δ*H* = −13 ± 1 kJ/mol
	Δ*S* = 58 ± 3 J/(mol·K)	Δ*S* = 16 ± 2 J/(mol·K)
	Δ*G* = −17.9 ± 0 kJ/mol	Δ*G* = −18.0 ± 0 kJ/mol
Li + BC15^a^	Δ*H* = −35 ± 7 kJ/mol	Δ*H* = 25 ± 5 kJ/mol
	Δ*S* = −95 ± 22 J/(mol·K)	Δ*S* = 95 ± 16 J/(mol·K)
	Δ*G* = −6.6 ± 0.1 kJ/mol	Δ*G* = −4.1 ± 0.2 kJ/mol
Li + BC18^a^	Δ*H* = −22 ± 3 kJ/mol	Δ*H* = 9 ± 2 kJ/mol
	Δ*S* = −50 ± 9 J/(mol·K)	Δ*S* = 47 ± 6 J/(mol·K)
	Δ*G* = −7.3 ± 0 kJ/mol	Δ*G* = −5.3 ± 0.1 kJ/mol

	temperature range (278–338 K)
U + BC15^a^	Δ*H* = −5.5 ± 2.9 kJ/mol
	Δ*S* = 61 ± 10 J/(mol·K)
	Δ*G* = −23.8 ± 0 kJ/mol
U + BC18^a^	Δ*H* = −3.6 ± 2.8 kJ/mol
	Δ*S* = 70 ± 9 J/(mol·K)
	Δ*G* = −24.5 ± 0 kJ/mol

a These data were cited from our previous literature.^3^ Δ*G* values at 298 K were described.

The maximum adsorption capacity of V in seawater was calculated by dividing the 12 mol (or 14 mol) of benzo-15-crown 5-ether (or benzo-18-crown 6-ether) on BC15 (or BC18) by the weight of the BC15 resin (or BC18 resin) on BC15 (or BC18), based on the assumption that the reaction of V against the 12 (or 14) adsorption points per unit of a crown ether ring proceeded. As a result, it was found that the obtained and calculated maximum adsorption capacities of V are 8.3 × 10^–2^ [mol/g of BC15 (calculated value)], 3.0 × 10^–5^ [mol/g of BC15 (experimental value)], 9.1 × 10^–2^ [mol/g of BC18 (calculated value)], and 4.0 × 10^–5^ [mol/g of BC18 (experimental value)], respectively. The results indicate that the adsorption points of BC15 (or BC18) are partially occupied by other elements in seawater. Hence, the chromatographic process is absolutely required to obtain the pure V, U, and Li salts. When this process was used, it was found that the mutual separation of V, U, and Li from Na is possible (see Figure S4).

**Figure 9:**
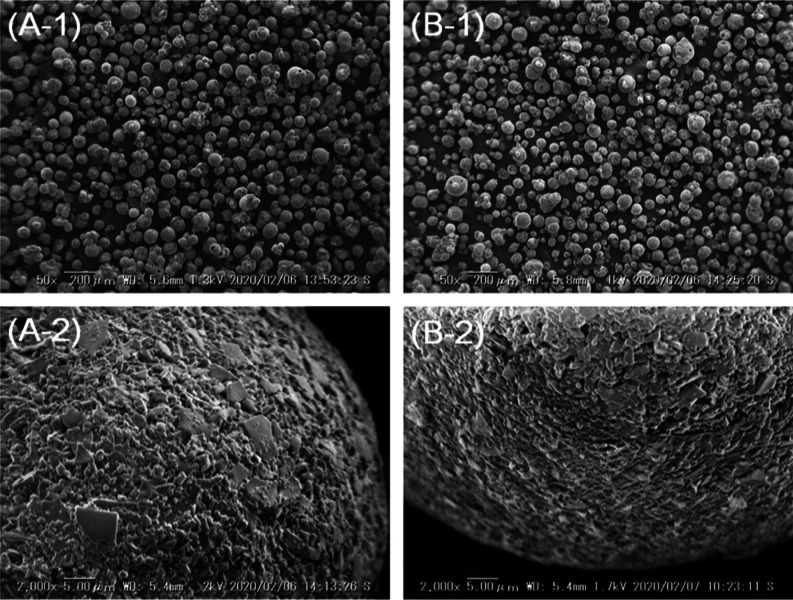
SEM images of BC15 and BC18 adsorbents. (A-1) BC15 after test, M = 50×, (A-2) BC15 after test, M = 2000×, (B-1) BC18 after test, M = 50×, and (B-2) BC18 after test, M = 2000×.

BC15 (or BC18) was rinsed by following the sequence of mobile phases (see Figure 2) after chromatography experiments in Figure 7. Then, BC15 (or BC18) recovered from the column was dried for a day in a vacuum dry oven at 60 °C until the weight of BC15 (or BC18) becomes constant. The change of the particle shape of BC15 (or BC18) was not observed entirely before and after experiments (see Figure 9). We unsuccessfully attempted to observe the chemical bonds between V and BC15 (or BC18) using FT-IR spectroscopy (IRAffinity-1S, Shimadzu). This is primarily caused by the wide overlap between the Si–O–Si stretching vibrations stemming from the ring structure of silica in the range from 1080 to 1050 cm^–1^ and the −C–O–C– stretching vibrations ranging from 1150 to 1070 cm^–1^ in the structure of crown ether rings (or −C–OH stretching vibrations ranging from 1040 to 1150 cm^–1^ in the structure of bisphenol A).

**Figure 10:**
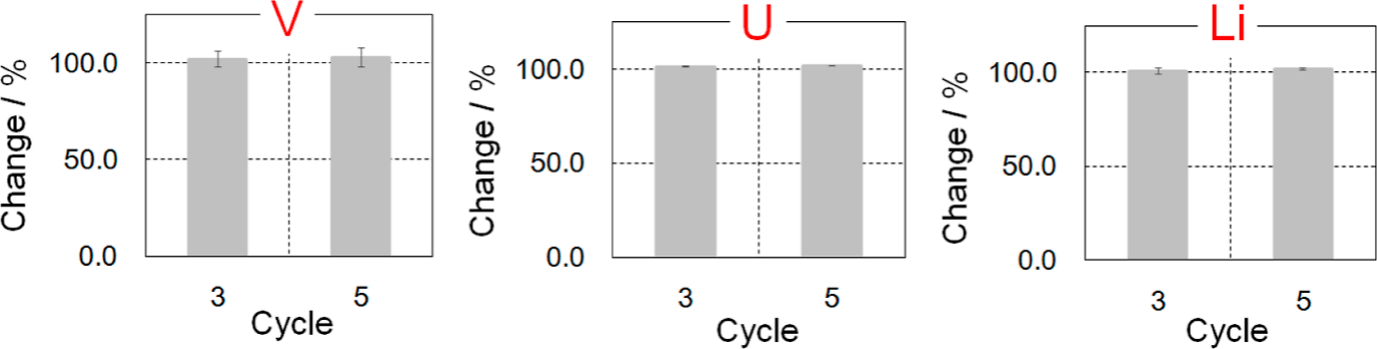
Change of adsorption ability of V, U, and Li after repeated experiments.

Finally, the durability of BC15 was evaluated from the viewpoint of industrial applications. In short, the aforementioned chromatography experiment was repeatedly performed to confirm whether V, U, and Li in seawater were stably and mutually separated or not. In addition, the values related to the thermodynamic stability of BC15 (or BC18), that is, the hardness (η), were supplementally obtained by the DV-Xα MO calculation; the computational details of this method have been described elsewhere.^67−70^ As a result, it was found that there is little difference between their η values (see Figure 1) and the V, U, and Li recovery performances do not decrease at all after at least five cycle tests. This indicates that these findings will be useful for developing a simple chromatographic technology to simultaneously recover V, U, and Li from seawater (see Figure 10).

## Conclusions

The use of BC15 and BC18 for simultaneous recovery of V, U, and Li from seawater has been suggested, and the adsorption and desorption behavior of these elements on BC15 (or BC18) has been widely checked in various types of aqueous solutions over a wide temperature range. In this result, it was noted that BC15 and BC18 have sufficient adsorption ability to simultaneously recover V, U, and Li from seawater. In addition, it was defined that the obtained *K*_d_ values of V decrease with an increase in [HCl]_T_, indicating that the chemical species of V (H_2_V_4_O_13_^4–^, V_4_O_12_^4–^, V_5_O_15_^5–^, and V_3_O_9_^3–^) dramatically change into the other V species (V^3+^, VO^2+^, and VO_2_^+^) in a 1.0 M HCl solution, and the complexation reactions between BC15 (or BC18) and the initial V structures are canceled. Besides, it was reasonably shown that the adsorption mechanism is the path through the electrostatic interaction between V (H_2_V_4_O_13_^4–^, V_4_O_12_^4–^, V_5_O_15_^5–^, and V_3_O_9_^3–^) and the −C–O–C– single bond in BC15 and BC18 (or the −C–OH single bond in BC15 and BC18). The difference in the *K*_d_ values of V (H_2_V_10_O_28_^4–^ and H_3_V_10_O_28_^3–^), U (UO_2_^+^ and UO_2_Cl^+^), and Li (Li^+^) on BC15 (or BC18) was clearly observed in a 1.0 × 10^–2^ M HCl solution. This implied that the mutual separation of V, U, and Li is possible by a chromatographic technique. Hence, the chromatography experiments of V, U, and Li on BC15 and BC18 were carried out using flowing seawater, 1.0 × 10^–2^ M HCl, and 1.0 M HCl in sequence and this hypothesis was validated. As a result, it was revealed that the first peak of V is separated from the plateau of Li and the first and second peaks of U in the case of the BC15 system. The recovery ratios of V and U were more than 80%. On the other hand, entirely overlapping chromatograms were obtained in the case of the BC18 system, and the recovery ratios of V and U were much lower. In short, the separation efficiency of V with BC15 was more pre-eminent than that of BC18. Furthermore, the maximum V adsorption capacity for BC15 (or BC18) was compared with those in previous reports. The obtained result showed that BC15 (or BC18) does not have a better adsorption capacity but has superior ability in the simultaneous recovery of V, U, and Li from seawater. Based on these results, the durability of BC15 was finally evaluated from the viewpoint of industrial applications. Concretely, the aforementioned chromatography experiment was repeatedly carried out to check whether V, U, and Li in seawater were stably and mutually separated or not. The obtained results showed that the recovery performances of V, U, and Li in seawater do not decrease at all after at least five cycle tests.

This achievement will be valuable for developing a practical chromatographic technology to recover simultaneously V, U, and Li from seawater. In the near future, its technology may become a catalyst to create novel valuable resources for resourceless countries facing the sea. Furthermore, our results have important implications for chemically understanding the simultaneous recovery of elements with complicated chemical forms from natural water. Especially, its interpretation may be useful for the clarification of other adsorption interactions except for the adsorption points of typical functional groups.

## Supporting Information Available

Distribution diagrams of V(III), V(IV), breakthrough curve of V(V), and chromatograms of V, U, Li, and Na (PDF)
