# Supplementary material for: Chromatographic Purification of Lithium, Vanadium, and Uranium from Seawater Using Organic Composite Adsorbents Composed of Benzo-18-Crown-6 and Benzo-15-Crown-5 Embedded in Highly Porous Silica Beads
Source: ACS Omega. 2022 Jul 27;7(31):27410–21. doi: 10.1021/acsomega.2c02427 (PMC9366790; doi:10.1021/acsomega.2c02427)
Supplement: Supplementary file 1 — ao2c02427_si_001.pdf [file ao2c02427_si_001.pdf]

Supporting Information

Chromatographic Purification of Lithium, Vanadium, and Uranium from Seawater Using Organic Composite Adsorbents Composed of Benzo-18-Crown-6 and Benzo-15-Crown-5 Embedded in High-Porous Silica Beads

Yu Tachibana[a*], Tomasz Kalak[b], Masahiro Tanaka[c]

[a]*Department of Nuclear System Safety Engineering, Graduate School of Engineering, Nagaoka University of Technology, 1603-1, Kamitomioka-machi, Nagaoka-shi, Niigata 940-2188, Japan*

[b]*Department of Industrial Products and Packaging Quality, Institute of Quality Science, Poznań University of Economics and Business, Niepodległości 10, Poznań 61-875, Republic of Poland*

[c]*National Institute for Fusion Science, 322-6, Oroshi-cho, Toki-shi, Gifu 509-5292, Japan*

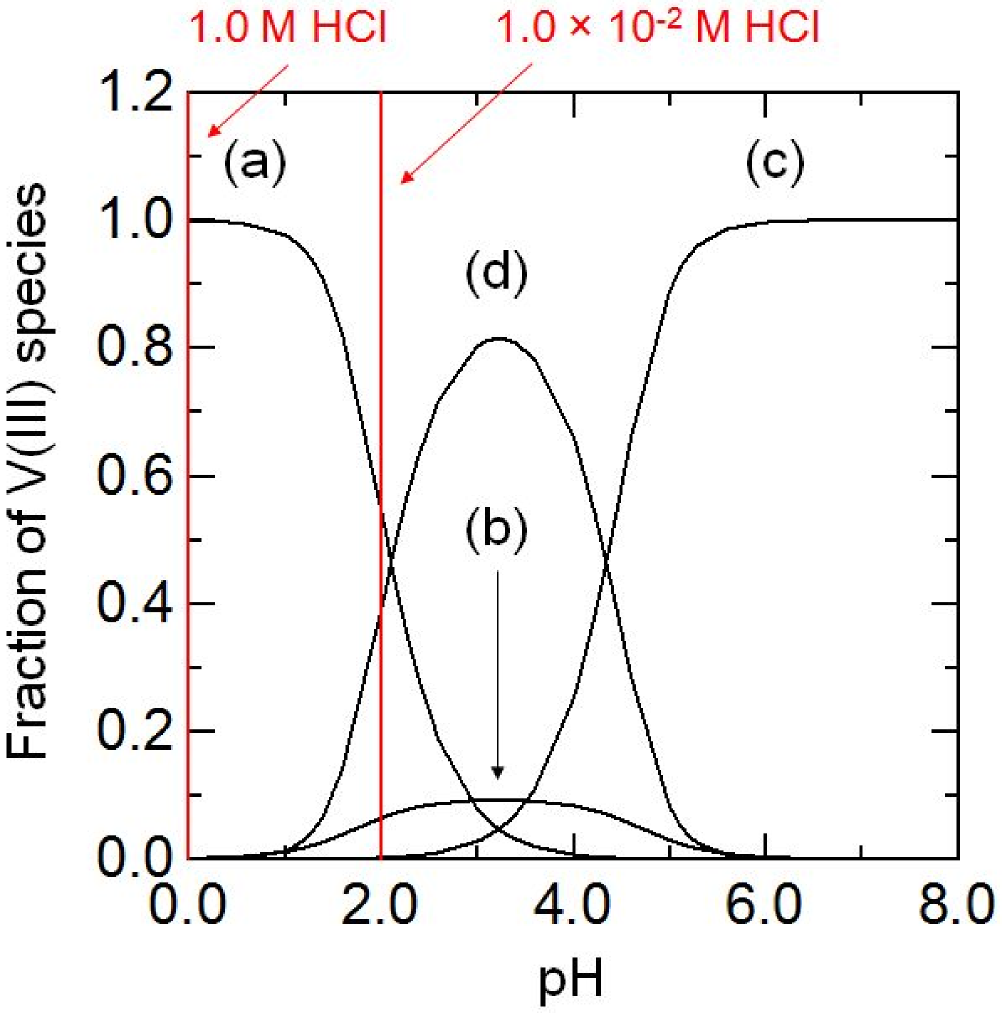

Figure S1. Distribution diagram of V(III) species as a function of pH. The stability constants between V^{3+}, VOH^{2+}, VO^{+}, $V_2(OH)_2^{4+}$ and H^{+}, H_2O are summarized by Israel and Meites in 1985.[61] (a): V^{3+}, (b): VOH^{2+}, (c): VO^{+}, (d): $V_2(OH)_2^{4+}$. The effect by the difference of their ionic strengths is not considered.

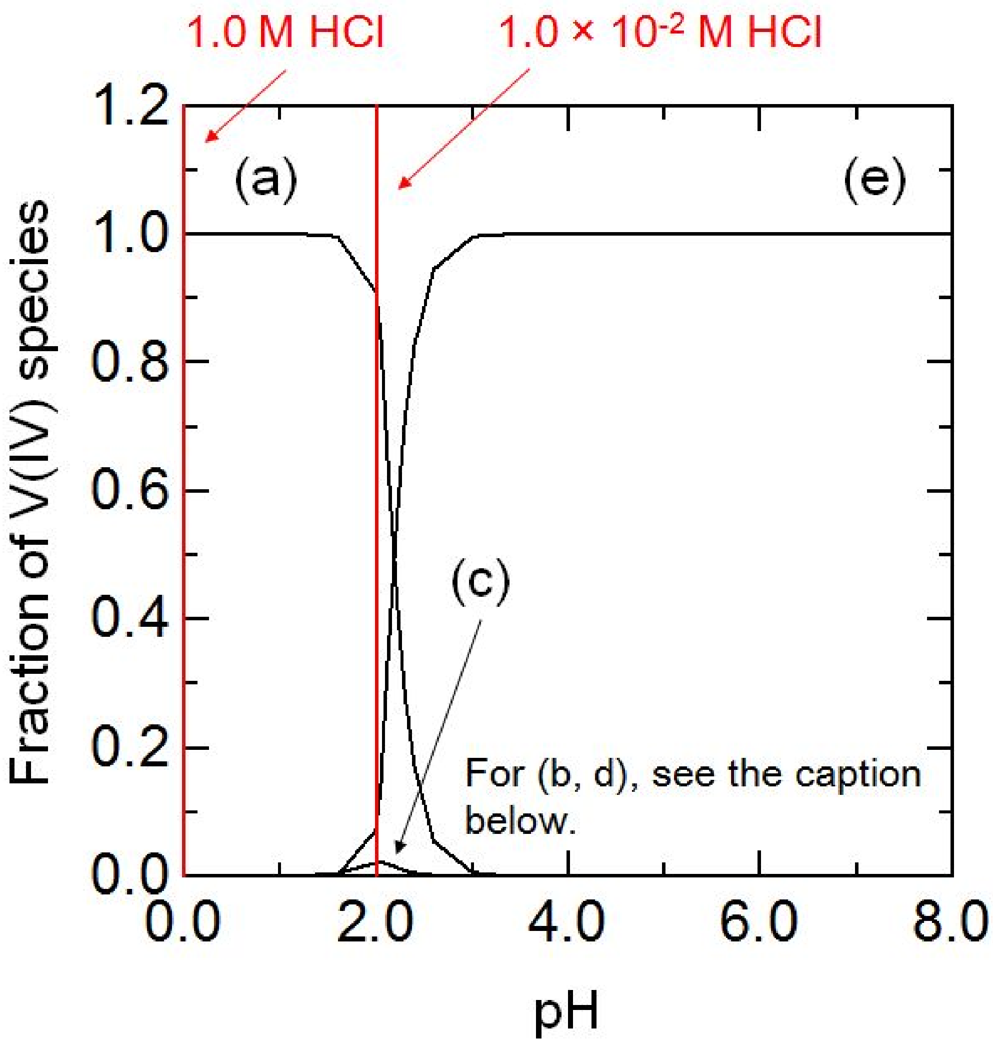

Figure S2. Distribution diagram of V(IV) species as a function of pH. The stability constants between VO^{2+}, $VOOH^{+}$, $(VOOH)_2^{2+}$, $HV_2O_5^{-}$, $V_4O_9^{2-}$, and H^{+}, H_2O are summarized by Israel and Meites in 1985.[61] (a): VO^{2+}, (b): $VOOH^{+}$, (c): $(VOOH)_2^{2+}$, (d): $HV_2O_5^{-}$, (e): $V_4O_9^{2-}$. With respect to the fraction of other species, (b): $VOOH^{+}$, (d): $HV_2O_5^{-}$ are excluded because of the small fraction of their V(IV) species. The effect by the difference of their ionic strengths is not considered.

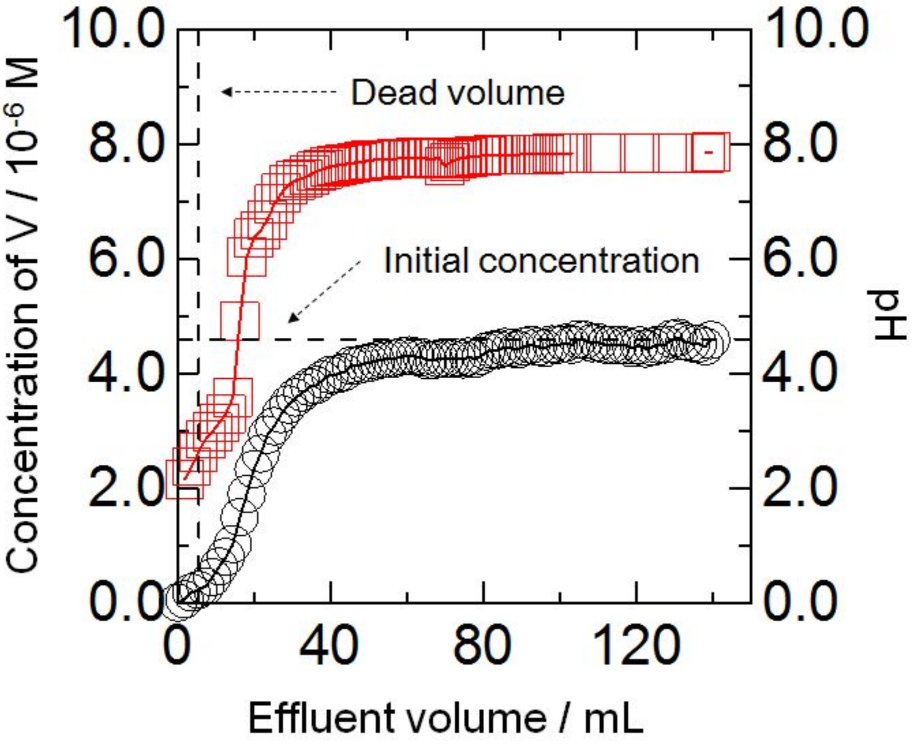

Figure S3. Breakthrough curve of V(V) in seawater using BC18 at 298 K. Particle size = 100 - 250 mesh. ○: Concentration of V (black), □: pH value (red).

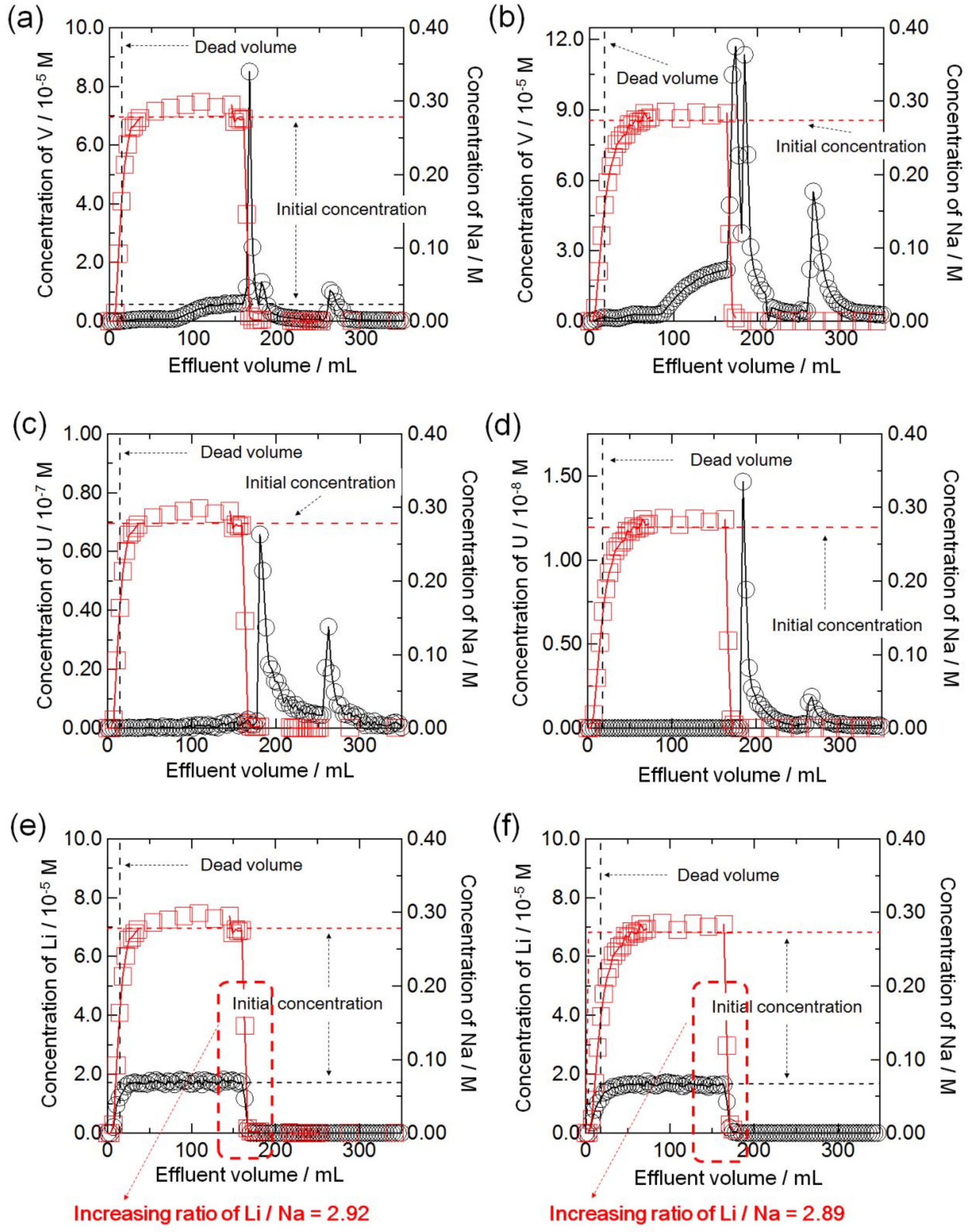

Figure S4. Chromatograms of V, U, Li, and Na using BC15 and BC18 at 298 K. Particle size = 100 - 250 mesh. ○: concentration of V, U, or Li, □: concentration of Na (red). (a) V and Na with BC15. (b) V and Na with BC18. (c) U and Na with BC15. (d): U and Na with BC18. (e) Li and Na with BC15. (f) Li and Na with BC18.
